# Strategies to Target ADAM17 in Disease: From Its Discovery to the iRhom Revolution

**DOI:** 10.3390/molecules26040944

**Published:** 2021-02-10

**Authors:** Matteo Calligaris, Doretta Cuffaro, Simone Bonelli, Donatella Pia Spanò, Armando Rossello, Elisa Nuti, Simone Dario Scilabra

**Affiliations:** 1 Proteomics Group of Fondazione Ri.MED, Research Department IRCCS ISMETT (Istituto Mediterraneo per i Trapianti e Terapie ad Alta Specializzazione), Via E. Tricomi 5, 90145 Palermo, Italy; mcalligaris@Fondazionerimed.com (M.C.); sbonelli@fondazionerimed.com (S.B.); 2 Department of Pharmacy, University of Pisa, Via Bonanno 6, 56126 Pisa, Italy; doretta.cuffaro@farm.unipi.it (D.C.); armando.rossello@farm.unipi.it (A.R.); 3 Università degli Studi di Palermo, STEBICEF (Dipartimento di Scienze e Tecnologie Biologiche Chimiche e Farmaceutiche), Viale delle Scienze Ed. 16, 90128 Palermo, Italy; donatellapia.spano@community.unipa.it

**Keywords:** ectodomain shedding, ADAM17, iRhoms, metalloproteinases, TIMPs, TNF, EGFR

## Abstract

For decades, disintegrin and metalloproteinase 17 (ADAM17) has been the object of deep investigation. Since its discovery as the tumor necrosis factor convertase, it has been considered a major drug target, especially in the context of inflammatory diseases and cancer. Nevertheless, the development of drugs targeting ADAM17 has been harder than expected. This has generally been due to its multifunctionality, with over 80 different transmembrane proteins other than tumor necrosis factor α (TNF) being released by ADAM17, and its structural similarity to other metalloproteinases. This review provides an overview of the different roles of ADAM17 in disease and the effects of its ablation in a number of in vivo models of pathological conditions. Furthermore, here, we comprehensively encompass the approaches that have been developed to accomplish ADAM17 selective inhibition, from the newest non-zinc-binding ADAM17 synthetic inhibitors to the exploitation of iRhom2 to specifically target ADAM17 in immune cells.

## 1. Introduction

The proteolytic release of transmembrane proteins, the so-called ectodomain shedding, is a key mechanism in several biological processes, including cell-to-cell communication and immunity [1]. A number of intercellular mediators, such as the tumor necrosis factor α (TNF), are synthesized as transmembrane proteins that need to be proteolytically released from the cell surface in order to trigger cell signaling. While efficient ectodomain shedding is necessary to maintain tissue homeostasis, its deregulation results in detrimental effects on cell behavior. For instance, TNF is a pro-inflammatory cytokine that plays a crucial role in the regulation of immune responses, directing the immune system to promptly respond to invading pathogens. In contrast, dysregulated TNF production contributes to the pathogenesis of a variety of human diseases, including autoimmune disorders, cancer, neurodegenerative diseases and many others [2,3,4]. TNF has been considered a major drug target for several years, and the anti-TNF inhibitors, a group of biomolecules that are able to block the signaling cascade triggered by TNF, are still the best-selling drugs worldwide [5]. TNF was first cloned in 1984, and four years later, discovered to be a transmembrane protein that needs to be proteolytically released from the cell surface in order to elicit its pro-inflammatory potential [6,7]. Almost 10 years passed before the “deus ex machina” regulating this process was discovered, when membrane-tethered disintegrin and metalloproteinase 17 (ADAM17) was identified as the TNF converting enzyme (TACE) [8,9]. Since then, targeting ADAM17 for drug development and fighting inflammatory diseases seemed to be the natural consequence of its discovery. Nevertheless, this process has been much harder than expected and never fully accomplished. The first drugs targeting the activity of ADAM17, despite the huge expectations, have never made it into the clinics, as the side effects that they caused were more severe than the benefits [10]. Indeed, these inhibitors blocked the activity of many other metalloproteinases that share with ADAM17 a conserved catalytic domain, thus deregulating a vast number of physiological processes. In addition, ADAM17, which is ubiquitously expressed in human tissues, is nowadays known to be a multifunctional proteinase that releases over 80 different substrates, other than TNF [11]. Having a catalytic domain that is almost indistinguishable from that of its closest relatives, and having a similar multifactorial function, made it difficult, if not impossible, to target ADAM17 for drug development. Nevertheless, researchers succeeded in developing strategies for a selective inhibition of ADAM17 over other metalloproteinases. This review will encompass all the approaches that have been developed to reach a selective inhibition of ADAM17 since its discovery, especially in the context of inflammatory diseases. These approaches span from functionalizing small molecules or engineering endogenous inhibitors to increase their selectivity for ADAM17 to the development of inhibitory biomolecules that target ADAM17 ancillary domains and finally to exploiting ADAM17 specific essential regulators iRhoms.

## 2. Biology of ADAM17

### 2.1. Structure

**Figure 1:**
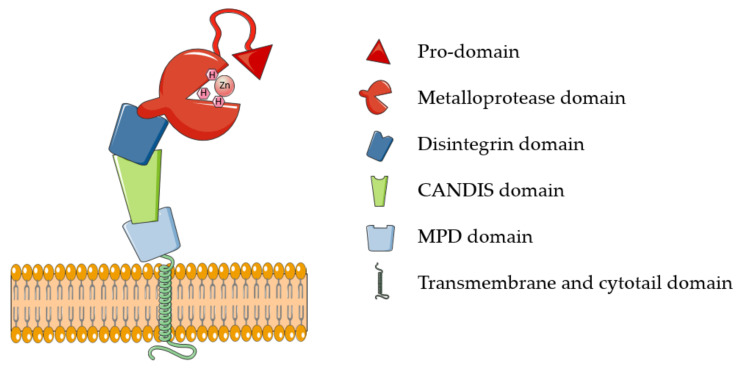
Schematic representation of ADAM17. ADAM17 comprises six different domains, here depicted with different shapes and colors: a pro-domain that constraints the proteolytic activity elicited by the metalloprotease domain. CANDIS: conserved ADAM seventeen dynamic interaction sequence; MPD: membrane proximal domain.

ADAM17 belongs to the family of membrane-tethered disintegrin and metalloproteases (ADAMs). These proteases are majorly involved in ectodomain shedding of cell membrane proteins. Around 30 ADAMs have been identified in mammals, but only half of them possess the characteristic metalloproteinase domain and proteolytic potential [12]. Additional to “active” ADAMs, the characteristic catalytic domain is highly conserved in all members of the metzincin superfamily, which comprises, among others, 23 members of the related matrix metalloproteases (MMPs) and 19 disintegrin metalloproteinases with thrombospondin domains (ADAMTSs). Metzincins, including ADAM17, use a Zn^2+^ ion for the catalysis that is coordinated to three histidines of the conserved binding motif HEXXHXXGXXH [10,13]. ADAM17 is synthesized as an inactive precursor containing an N-terminal pro-domain that constrains the enzyme activity through a Cysteine switch mechanism, which is common to the majority of metzincins [13]. Based on this, a pivotal cysteine contained in the conserved PRCGXPD motif coordinates the catalytic zinc, preventing it from coordinating water molecules and carry out the catalysis. Pro-domain removal and activation of ADAM17 involves the action of furin and takes place intracellularly within the secretory pathway [14]. In addition to the prodomain and catalytic domain, ADAM17 harbors ancillary domains for which physiological functions are still largely unknown: a disintegrin domain, which is involved in molecular interactions with other transmembrane proteins, including integrins; a membrane proximal domain (MPD) that regulates conformational changes of the enzyme; and a short stalk domain called CANDIS (Conserved Adam seventeeN Dynamic Interaction Sequence) (Figure 1). Proximal to the stalk region are a transmembrane domain, majorly involved in ADAM17 interaction with its essential regulators iRhom1 and 2, and an intracellular cytoplasmic domain whose physiological function is still unclear [11].

### 2.2. ADAM17 Function

ADAM17 was the first “sheddase” to be characterized. It mediates ectodomain shedding of different proteins, spanning from signaling molecules, such as cytokines, growth factors and their receptors, to adhesion molecules and endocytic receptors (Table 1) [11]. Thus, it is clear from its plethora of substrates that ADAM17 is involved in several biological processes, which continue to increase as long as novel substrates of the enzyme are discovered. For this reason, in this review, we will focus only on those physiological and pathological functions that have been characterized in vivo, especially in the context of inflammatory diseases.

### 2.3. Developmental Defects of ADAM17-Deficient Mice Are Majorly Due to Diminished EGFR Signaling

ADAM17 is ubiquitously expressed in mammals [15]. Its constitutive ablation is perinatally lethal in mouse, highlighting its crucial role in development. This is majorly driven by ADAM17’s ability to trigger EGFR signaling by shedding its ligands [15]. In support of this hypothesis, ADAM17 knockout (KO) mouse phenocopies ablation of the ADAM17 substrate TGFα in that mice are born with open eyes and die perinatally, suggesting that ADAM17-mediated shedding of TGFα and subsequent EGFR activation is the major pathway controlled by ADAM17 during development [15]. Due to the early mortality of ADAM17-deficient mice, in vivo functions of the enzyme and its role in disease have been elucidated by using conditional knockouts, or a hypomorphic ADAM17 mouse, which does not show developmental abnormalities and expresses reduced levels of ADAM17 transcripts and protein [16]. In addition to the clear developmental defects induced by a lack of ADAM17, ADAM17 conditional KO mice have shown additional phenotypes associated with impaired EGFR signaling. Mice lacking ADAM17 in keratinocytes have a normal epidermal barrier and skin morphology at birth but develop severe defects in epidermal barrier integrity soon after birth and develop chronic dermatitis in adulthood [17]. ADAM17 deficiency in chondrocytes leads to defects in endochondral ossification, so that mice have a significantly expanded zone of hypertrophic chondrocytes in the growth plate and retarded growth of long bones [18]. Defects in both maintenance of skin barrier and endochondral ossification are phenocopied by ablation of EGFR and rescued by TGFα, indicating for the ADAM17/EGFR axis a pivotal role in these processes [17,18]. ADAM17 conditional KO mice under control of Sox9, a transcription factor expressed by proliferating chondrocytes, exhibited defects in the skeletal architecture, with shorter long bones and prominent bone loss by controlling the regulation of IL-17 and GM-CSF, two essential mediators of chondrocyte differentiation [19].

### 2.4. Role of ADAM17 in Inflammation

The role of ADAM17 in inflammation became evident as soon as it was identified as the enzyme responsible for the release of soluble TNF. Since then, ADAM17 has been discovered to control the release of other proteins involved in this process, including IL-6R and L-selectin. This highlights the complexity of ADAM17 function in inflammation, which orchestrates not only one, but multiple key molecular pathways of the inflammatory process.

As a consequence of its central role in TNF release, ADAM17 inactivation in myeloid cells, or its temporal inactivation in the Mx1-Cre model—where ADAM17 is ablated in an interferon-dependent manner following injection of polyinosinic:polycytidylic acid (pIpC)—led to strong protection from endotoxin shock lethality [20]. By using chimeric mice in which bone marrow was depleted by irradiation and reconstituted with ADAM17-deficient fetal liver cells, Bruce Walcheck’s group identified L-selectin and TNFR1 and -2 as physiological substrates of ADAM17 and pioneered a comprehensive study on the role of ADAM17 in leukocytes [21,22]. They found that the chimeric mouse harboring ADAM17-deficient leukocytes had longer survival after *Escherichia coli*-mediated peritoneal sepsis, which was associated with a reduction in systemic proinflammatory cytokine levels, including TNF, and bacterial burden [23]. Furthermore, preventing the shedding of L-selectin, the chimeric mice displayed an augmented neutrophil recruitment and higher pathogens clearance [23].

Furthermore, the ADAM17 substrate IL-6R is the major mediator of IL-6 classic and trans-signaling pathways [24]. In the classic IL-6 signaling pathway, IL-6 binds to membrane tethered IL-6 receptor (IL-6R) and promotes resolution of inflammation by activation of STAT1 and STAT3 [25]. The classic IL-6 pathway is restricted to lymphocytes and hepatocytes, as these are the only cells that express IL-6R in the body. Despite being regulated by the same molecular mediators, IL-6 and IL-6R, the IL-6 trans-signaling pathway promotes activation of immune responses and it is associated with the development of several inflammatory diseases, including rheumatoid arthritis [26]. ADAM17 plays a key role in activating IL-6 trans-signaling pathway, as it sheds the ectodomain of IL-6R, both in vitro and in vivo [27,28]. Soluble IL-6R (sIL-6R) retains its ability to bind IL-6, preventing it from binding to the membrane-tethered form of the receptor. In contrast, the IL-6/sIL-6R complex binds to another membrane receptor, gp130, which is expressed on majority of cells [26].

### 2.5. Role of ADAM17 in Skin Homeostasis

The epidermis is the outermost layer of the skin and functions as a barrier to protect from water loss and infections from environmental pathogens. The epidermis undergoes continuous regeneration by a very organized process that includes terminal differentiation of keratinocytes. Within this process, called cornification, keratinocytes proliferate, detach from the basement membrane and form the cornified layer [29]. Other than forming a physical barrier to pathogens, keratinocytes guide the immune system to eliminate infected cells by initiating a well-orchestrated network of signaling. ADAM17 plays a fundamental role in both keratinocytes terminal differentiation and maintaining skin homeostasis. Ablation of ADAM17 in keratinocytes led to compromised epidermal barrier in mouse as a consequence of dysregulated keratinocytes terminal differentiation [17]. Ablation of EGFR in keratinocytes phenocopied these abnormalities, while ectopic application of TGFα to reversed the keratinocytes terminal differentiation and skin inflammation, clearly indicating that ADAM17 controls skin homeostasis by regulating the shedding of TGFα.

Murthy and colleagues discovered that ADAM17 controls communication between keratinocytes and immune cells by activating Notch signaling [30], which is involved in a variety of cell differentiation processes during embryonic and adult life [31]. ADAM-mediated cleavage of Notch receptors (called S2-cleavage) is a critical step in the activation of Notch signaling [32]. This process is activated by a number of Notch ligands, type 1 transmembrane proteins that are expressed on the surface of a signal-sending cell. Upon their binding to Notch, an ADAM-mediated cleavage of Notch allows the gamma-secretase to further process its transmembrane domain and release the intracellular domain, which, subsequently, translocates into the nucleus and activates target gene expression. An ADAM10 homologue was the first protease to be characterized as a Notch sheddase in Drosophila [33]. Later, ADAM10 and ADAM17 were uncovered to be major Notch sheddases in mammals, with ADAM10 having a prominent role in the ligand-dependent activation of Notch, and ADAM17 in its ligand-independent activation [32]. Other than forming a physical barrier to invading pathogens, keratinocytes play a critical role in recruiting immune cells to eliminate infected of damaged cells. ADAM17 regulates the keratinocytes-immune system crosstalk through Notch-signaling activation [30]. Ablation of ADAM17 in keratinocytes reduced Notch signaling. This, in turn, increased the production of the granulocyte macrophage colony stimulating factor (GM-CSF), a cytokine that promotes neutrophil proliferation and maturation, and thymic stromal lymphopoietin (TSLP), a cytokine that is known to play an important role in the maturation of T cell populations and involved in the onset of atopic dermatitis [34,35,36]. In agreement, keratinocyte-specific deletion of ADAM17 induced atopic dermatitis and myeloproliferative disease in mouse, which were reversed by transduction of active Notch (NICD) in the same cells [30].

## 3. ADAM17 Regulation

### 3.1. Transcriptional and Post-transcriptional Regulation

The activity of ADAM17 has to be finely tuned, and this occurs at several levels, including transcriptional and post-transcriptional regulation. ADAM17 upregulation is associated with a number of chronic inflammatory diseases, including arthritis and atherosclerosis, and malignancies [37,38]. Its expression can be regulated by transcription factors such as NF-kB and Elk-1 [39]. ADAM17 can be regulated by epigenetic mechanisms, including recruitment of the chromatin remodeling protein BRG1, which promotes alteration of chromatin structure through the histone demethylase KDM4 and leads to ADAM17 expression [40]. Furthermore, a number of miRNAs have been validated to downregulate ADAM17 expression and lower the release of its substrates, including TNF. The best characterized is miR-145 [41,42,43]. miR-145 directly binds to the ADAM17 3′-UTR, thereby reducing its expression [41]. As a consequence, miR-145 suppresses cancer progression [41,42,43]. In contrast, downregulation of miR-145 is associated with glioma cells, where proliferation, migration and invasion are due to enhanced ADAM17 levels and activation of EGFR pathway. Similarly, miR-124 blocks ADAM17 expression by binding its 3′-UTR leading to reduced TNF-α release and IL-6 production [44]. In addition, miR-152 and miR-326 target ADAM17 and suppress progression of non-small cell lung cancer and lung adenocarcinoma, respectively [45,46]. Nevertheless, an increase in ADAM17 gene expression is often not followed by the same increase in ADAM17 activity. Indeed, overexpression of ADAM17 in mice does not enhance its shedding activity in vivo and the pathology of ADAM17-assciated diseases. Thus, it is clear that besides regulation of its mRNA levels, a well-organized web of regulators, cofactors and protein inhibitors ensures a correct spatio-temporal modulation of ADAM17 function, which is essential to maintain tissue homeostasis.

### 3.2. Removal of the Pro-domain

ADAM17 is translated into the ER as an inactive protease comprising a pro-domain that constraints its activity during the secretory pathway [47]. This prevents the enzyme from cleaving proteins in an unspecific manner before reaching the Golgi, where its removal occurs by action of a pro-protein furin convertase [48]. The importance of the pro-domain in the regulation of ADAM17 is demonstrated in vivo by a cancer-associated point mutation that falls within its sequence (R177C) [49]. A further analysis in vitro shows that this mutation impaired ADAM17 proteolytic activity and its tracking to the cell membrane in a similar manner as a lack of the entire pro-domain affected its maturation [49].

### 3.3. Structural/Conformational Changes

#### 3.3.1. Membrane Interactions

The non-catalytic domains of ADAM17 play a crucial role in the regulation of its activity, as modifications of these domains mediate the reversible conformational change between an “off” state, in which the enzyme is inactive, to an “on” state, in which the enzyme is ready to cleave its substrates. Evidence suggests that the membrane proximal domain (MPD) and the CANDIS can modulate ADAM17 conformation and activity by forming electrostatic interactions with the cell membrane. While ADAM17 remains in an inactivated state in cholesterol-rich microdomains of the membrane, regions rich in phosphatidylserines promote its activation [50,51]. A short positively charged motif within the MPD (R625-K626-G627-K628) is crucial in this process, in that it binds to phosphatidylserines in the outer layer of the membrane, thereby keeping ADAM17 in the “on” state [52]. Phosphatidylserines are commonly present in the inner layer of the membrane and exposed towards the extracellular milieu in apoptotic cells. Interestingly, the same stimuli that lead to ADAM17 activation (which will be discussed in detail in Section 4.5) are also known to promote flipping of phosphatidylserines in the outer layer of the membrane, thus suggesting a model in which specific stimuli induce activation of the enzyme and membrane rearrangement that is necessary to keep the enzyme in the “on” state [53]. Centrality of the phosphatidylserine binding motif in ADAM17 function was further demonstrated in vivo. Indeed, Veit and colleagues manipulated a mouse by CRISPR/Cas9 in order to delete the small cluster of cationic amino acids R625/K626/G627/K628 that comprise the phosphatidylserine-binding motif of ADAM17 (referred to as the ADAM17^3x/3x^ mouse). Interestingly, the phenotype of resulting mice was more severe than that shown by deletion of ADAM17. While a lack of ADAM17 is perinatally lethal, deletion of the phosphatidylserine-binding motif led to embryonical lethality, with no embryos obtained beyond E16 [54].

#### 3.3.2. Protein Disulfide Isomerase

Another crucial molecular switch able to modulate the “on/off” state of ADAM17 is represented by the protein disulfide isomerase (PDI). This enzyme is responsible for maintaining ADAM17 in an inactive form, and consequently, its downregulation markedly enhanced ADAM17 activation [55]. PDI directly interacts with the MPD, where it catalyzes an isomerization of two disulfide bridges, thus keeping ADAM17 in an “off” conformation. Again, levels of PDI are rapidly downregulated by the same stimuli, leading to ADAM17 activation, including activation of PKC.

#### 3.3.3. Integrins

Several ADAMs are known to interact with integrins, and ADAM17 was found to bind to integrin α5β1 through its disintegrin domain. This interaction affects ADAM17-mediated cell adhesion and cell migration, indicating that this integrin can modulate the activity of ADAM17 [56].

#### 3.3.4. Glycosylation

Glycosylation of a recombinant soluble form of ADAM17 has been reported to affect activity and inhibition of the enzyme. ADAM17 was expressed in mammalian cells, where it was heavily glycosylated, or in insect cells, where it was less glycosylated [57]. More glycosylated ADAM17 had a lower k_cat_ against synthetic substrates generated on its TNF cleaving sequence, suggesting that glycosylation may play a role in the regulation of ADAM17 activity. However, the physiological relevance of this finding has to be determined, as the enzyme can function in a very different manner when tethered to the membrane and in its physiological cellular environment.

#### 3.3.5. ADAM17 Shedding and Soluble ADAM17 Degradome

The ectodomain of ADAM17 can be shed itself as a consequence of a cleavage mediated by ADAM8 [58]. Soluble ADAM17 was demonstrated to still be active, but its substrate repertoire drastically changed. Of all known ADAM17 substrates, the analysis performed by Scharfenberg and colleagues identified only CSF1 as a protein cleaved by soluble ADAM17. In contrast, soluble ADAM17 acquires the capability to cleave a number of extracellular matrix (ECM) proteins, including thrombospondin-4 and type-4 collagen, which canonically are not degraded by the metalloproteinase [58].

Other than its shed ectodomain, soluble ADAM17 can be found in the extracellular medium associated with extracellular vesicles [59,60]. It has recently been found that ADAM17 can diverge from its canonical maturation pathway, involving translocation from ER to the Golgi, and be packed into EVs for unconventional secretion. The catalytic domain of EV-associated ADAM17 protrudes extracellularly, and it can elicit its proteolytic potential, as it was shown to mediate shedding of TGFα and amphiregulin [60]. Functional consequences in vivo of this regulatory mechanism are still unknown, but it is clear that ADAM17 activity can be distributed to more distant cells by hitchhiking extracellular vesicles.

### 3.4. iRhoms

#### 3.4.1. ADAM17 Trafficking and Maturation

**Figure 2:**
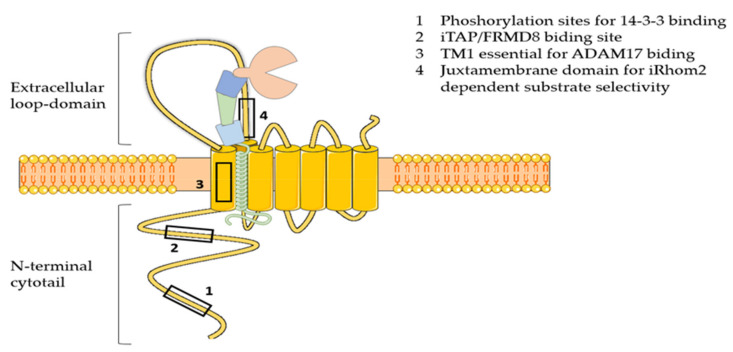
Schematic representation of iRhom2. iRhom2 comprises 7 transmembrane domains (TM1-7), the first of which is crucial for the interaction with ADAM17. In addition, iRhom2 exhibits a number of unique features compared to rhomboids that are essential for regulating the activity of the protease: 1) the N-terminal cytotail, which contains the phosphorylation sites for binding of 14-3-3 proteins and the iTAP/FRMD8 binding site; 2) the large extracellular loop domain between the first and the second transmembrane domain, which harbors a sequence that is essential for iRhom2-dependent substrate selectivity.

Two inactive cognates of rhomboid proteases, namely iRhom1 and iRhom2, have been discovered as essential regulators of ADAM17 maturation and activity [61]. Differently from rhomboids, these seven membrane-spanning pseudoproteases have lost the catalytic motif GxS during evolution, and acquired essential features for ADAM17 regulation, including a long N-terminal cytotail that is crucial for the enzyme activation, and a large loop between the first and second transmembrane domain that is needed for ADAM17 selectivity towards specific substrates (Figure 2) [61]. iRhoms and ADAM17 form a tight complex in the ER. Recently, it has emerged that ADAM17 is necessary for stabilization of iRhom2, which gets degraded in the absence of the protease [62]. This evidence suggests that iRhoms can be considered as the regulatory subunits of an ADAM17 proteolytic complex, rather than orthodox modulators of the protease activity. After forming a complex, iRhoms guide ADAM17 trafficking from the ER to the cell surface, passing through the Golgi, where the pro-domain of the enzyme is removed and its proteolytic potential liberated [63,64]. When iRhoms are ablated, ADAM17 is retained in the ER and its maturation is impaired [65,66]. In vivo analysis showed that iRhom1 and 2 have redundant ability in supporting ADAM17 maturation [66]. When either iRhom is lacking, the other one is able to compensate. In agreement, Li and colleagues have shown that iRhom1 or iRhom2-deficient mice have no phenotype, whereas iRhom1 and 2 double knockout mice phenocopy ADAM17-deficient mice in that they are born with open eyelids, present heart valve defects and die soon after birth [66]. In contrast, iRhoms-deficient transgenic mice generated by Christova et al. displayed a more severe phenotype. In this case, rather than being normal, iRhom1-deficient mice have brain and heart defects, and die early [65]. In addition, iRhom1 and 2 double knockout mice die in utero at E9.5, rather than perinatally, as it is shown by Li et al. This suggests that the function of iRhom1 and 2 is not fully redundant, and that they must have additional functions to guiding ADAM17 maturation. The discrepancy between these two mouse models may arise from the strategy used to ablate iRhom1 and 2, as has been reported by Hosur et al. [67]. This work demonstrated that the complete deletion of iRhom1 coding sequence leads to the severe phenotype reported by Christova et al. In contrast, the strategy used by Li et al. of deleting the sequence spanning from exon 4 to 11, where the canonical starting codon resides, leads to the expression of a truncated form of iRhom1 that originates from an alternative starting codon. This iRhom1 variant is shown to retain some functions of the wild-type protein, including stimulating ADAM17-dependent shedding of amphiregulin, and allows mice to grow up normally. Although this study provided insight into the discrepancy between the two iRhom1-deficient mouse models so far generated, the biochemical characterization of the iRhom1 variant, in our opinion, is not conclusive, and functions retained or acquired by this truncated variant should be investigated more carefully.

#### 3.4.2. ADAM17 Activation

ADAM17, similarly to other canonical sheddases, cleaves its substrates at a fixed distance close to the cell membrane [1]. It has been historically considered an activated sheddase, which releases its substrates in response to specific stimuli. GPCRs, including muscarinic receptors and protease-activated receptor 1 (Par1) [68,69], are examples of physiological activators of ADAM17. Protein kinase C (PKC) plays a central role in the activation of ADAM17, and the phorbol ester PMA (phorbol 12-myristate 13-acetate), which induces phosphorylation of the kinase, is commonly used to trigger ADAM17 activity [70]. ADAM17 has several phosphorylation sites within the cytoplasmic tail, whose physiological function has been under debate. Some studies have reported a role for these phosphorylation sites in the regulation of ADAM17. For instance, PMA induced ADAM17 phosphorylation on threonine 735 by mitogen activated protein (MAP) kinase with a subsequent increase in cleavage of the transmembrane TrkA neurotrophin receptor [71]. In addition, Elliott and colleagues demonstrated that angiotensin II requires the ADAM17 cytotail, and specifically phosphorylation at tyrosine 702 by Src, in order to activate HB-EGF shedding [72]. In contrast, others have reported that the cytoplasmic tail is dispensable for ADAM17 activation [73]. The discovery of iRhom2 tipped the balance in favor of the latter model. Based on this, the transmembrane domain of ADAM17, which had been proven essential for its rapid activation, interacts with the first transmembrane domain of iRhom2 [74]. Disruption of this interaction abolished PMA-mediated activation of the metalloproteinase. Structural modeling was used to identify transmembrane residues of ADAM7 that are relevant in the interaction with iRhom2 (Figure 2) [74]. Point mutation of such residues diminished ADAM17-mediated shedding. Interestingly, these mutations did not affect iRhom1-dependent shedding of ADAM17, suggesting that iRhom1 and iRhom2 can transduce external stimuli and activate ADAM17 by a different mechanism. Furthermore, rather than a direct phosphorylation of ADAM17, it is currently believed that phosphorylation of the N-terminal cytotail of iRhom2 plays a central role in transducing external stimuli leading to ADAM17 activation [75,76]. Indeed, two independent groups have recently shown that ADAM17 activation stimuli, including PMA and GPCRs, initiate a signaling cascade culminating in the phosphorylation of iRhom2 cytotail by MAP kinases. Based on this model, iRhom2 rains in ADAM17 function until the phosphorylated cytotail recruits 14-3-3 proteins. This, in turn, leads to a conformational change in iRhom2 that allows ADAM17 to cleave its substrates [77,78]. In addition to regulating the protease activation, iRhoms can enhance the stability of ADAM17, preventing it from lysosomal degradation [77,78]. A membrane anchoring protein, called FRMD8, has been identified by interactomics and proven essential in this process [78]. Furthermore, iRhom1 and iRhom2 can address the activity of ADAM17 towards specific groups of substrates. Maretzky and colleagues found that some substrates can be released in response to PMA, when ADAM17 is in a complex with either iRhom1 or iRhom2 [79]. In contrast, some proteins can be released by ADAM17 in an iRhom2-dependent manner, and iRhom1 is not able to compensate. The juxtamembrane domain within the large loop between TMD1 and TMD2 of iRhom2 has been proven to play a central role in this process [80]. Given that only a limited number of prototypical ADAM17 substrates have been analyzed in these studies so far, no proteins whose shedding is only dependent on iRhom1 have been identified. A systematic shedding analysis of ADAM17 substrates would be required to evaluate whether proteins that are specifically shed in an iRhom1-dependent manner exist. Such an analysis would be useful to uncover molecular determinants that render shedding of ADAM17 substrates as iRhom1- or iRhom2-dependent. Finally, negatively charged residues within the stalk region of ADAM17 substrates emerged as pivotal in directing their shedding, as they confer shedding resistance to a number of ADAM17 substrates [81]. Although not proven yet, given the crucial role of iRhoms in ADAM17 activity, these results suggest that iRhoms, additionally to ADAM17, may take contacts with its substrates, which can be disrupted by negatively charged residues within their stalk region.

In conclusion, iRhoms emerged as the regulatory subunit of ADAM17/iRhom proteolytic complexes. iRhoms drive trafficking and maturation of the enzyme, regulate its activity in response to stimuli, direct its proteolytic activity towards specific groups of substrates and, potentially, recruit its substrates and modulate the accessibility of the enzyme to their cleavage sites.

#### 3.4.3. TIMP-3 Inhibition

Once at the cell surface, the activity of ADAM17 can still be regulated by the tissue inhibitor of metalloproteinase 3 (TIMP-3). TIMP-3 belongs to a family of four protein inhibitors (TIMP-1 to -4) that inhibit metalloproteinases with a different selectivity. Among them, TIMP-3 has the broadest inhibitory profile, as it is the only TIMP able to inhibit members of ADAMs, including ADAM17, ADAMTSs and MMPs [82]. The crystal structure of TIMP-3 in complex with ADAM17 has been determined [83]. Similar to the other TIMPs, TIMP-3 has a “wedge shape” that perfectly complements the active-site cleft of the enzyme, and a highly conserved Cys1-X-Cys3 structure at the N-terminal region, which is crucial for inhibiting metalloproteinases. TIMP-3 inserts into the active site cleft of ADAM17 and coordinates the proteolytic Zn^2+^ ion with its Cys1. This, in turn, displaces from the water molecule needed for peptide bond hydrolysis from the zinc. What really characterizes the specificity of TIMP-3 binding to ADAM17 is the interactions between Thr2 of TIMP-3 and the so-called S1′ specificity pocket of the enzyme. Indeed, Thr2 is not conserved in TIMP-2 and -4 binding site, where a serine occurs. Furthermore, the Phe34 and the two adjacent Leu67 and Leu94 play a pivotal role in the specific inhibition of ADAM17 by TIMP-3, as their side chains make favorable interactions with a unique hydrophobic groove of the TACE surface. The effects of ADAM17 regulation by TIMP-3 have been evaluated in vivo. Loss of TIMP-3 in mice leads to dysregulated TNF release and subsequent increase of MMP and ADAMTS activity, thereby promoting increased cartilage breakdown in both models of inflammatory and surgically-induced arthritis [84,85], while articular injection of TIMP-3 ameliorated the pathology in a rat meniscal tear model of osteoarthritis [86]. Furthermore, ablation of TIMP-3 and subsequent dysregulated ADAM17 activity and TNF release promoted insulin resistance and hepatosteatosis [87,88]. Conversely, by targeting the ADAM17/TNF axis, overexpression of TIMP-3 in mouse macrophages reduced adipose inflammation, insulin resistance and nonalcoholic fatty liver disease, other than reducing atherosclerotic plaques in a mouse model of atherosclerosis [89,90,91]. Because of its unique ability to inhibit ADAM17 among the four mammalian TIMPs, TIMP-3 has been considered as a valuable potential therapeutic target for the treatment of inflammatory diseases, and several approaches to enhance its selectivity for ADAM17 over MMPs have been pursued to develop efficient therapies with low risks of mechanism-based side effects.

## 4. ADAM17 in Disease

### 4.1. ADAM17 in Rheumatoid Arthritis

TNF levels are increased in most chronic inflammatory diseases, supporting the involvement of ADAM17 in their progression, including rheumatoid arthritis (RA) [92]. The importance of TNF in RA was initially proposed based on a study aiming to identify highly expressed cytokines at the local site of the disease, the synovium [93]. Its blockage by neutralizing antibodies dampened production of other pro-inflammatory mediators, including IL-1, and ameliorated the pathology in animal models of the disease [94]. Since then, anti-TNF inhibitors have been largely used for the treatment of RA and other inflammatory diseases and become blockbuster drugs, giving profits of over 20 million dollars worldwide in 2020 [5]. In addition to the clinical outcome of anti-TNF therapy, a number of preclinical studies have been carried out to validate the role of the ADAM17/TNF axis. Ablation of ADAM17 in myeloid cells is protective against rheumatoid arthritis to a similar extent as the ablation of TNF [95]. Interestingly, a transgenic mouse carrying an uncleavable form of TNF and, therefore, lacking exclusively the soluble form of TNF (solTNF) but not its transmembrane-bound form (tmTNF), was better protected against chronic inflammation than a TNF-deficient mouse [96]. In agreement, molecules able to block solTNF, but not tmTNF, were more efficient than anti-TNF agents in ameliorating inflammatory diseases [97]. Indeed, additional studies have displayed differential functions for the two forms of TNF. SolTNF or tmTNF have differential affinities for TNF receptors (TNFR1 and TNFR2) in mouse and trigger opposite cell responses [98]. Ablation of one or the other TNF receptor leads to diverse outcome in disease. SolTNF preferentially binds to TNFR1, which is ubiquitously expressed [99]. This event induces pro-inflammatory signaling through the activation of NF-kB, a transcriptional activator that induces the expression of several pro-inflammatory genes, including cytokines and chemokines [100]. In agreement, TNFR1-deficient mice show reduced inflammatory responses and arthritis [95]. Diversely, tmTNF preferentially binds to TNFR2, which has anti-inflammatory and protective properties [101]. Knockout studies in mice have shown that ablation of TNFR2 leads to an increase of solTNF in the plasma and enhanced TNFR1-mediated inflammation, suggesting that TNFR2 plays a major role in controlling inflammation by extinguishing TNF-dependent signaling [101]. These results highlighted a role for ADAM17 as a molecular switch that is able to shift immune responses from an anti-inflammatory to a pro-inflammatory state. It is evident that selective inhibition of ADAM17-dependent cleavage of TNF would favor an anti-inflammatory response and represent a drug target that potentially may lead to the development of therapies more effective than anti-TNF.

As discussed above, ADAM17 governs the IL6R trans-signaling pathway that, additional to the TNF pathway, drives the overexpression of inflammatory genes and sustains progression of RA [102]. A soluble variant of gp130, the signaling receptor for the IL6/IL6-R complex, acts as a decoy receptor and counteracts IL6 trans-signaling and STAT3 activation, thereby ameliorating the pathology in a murine model of the disease [103]. Tocilizumab, the commercial name of anti-IL-6R antibodies, was approved for treatment of rheumatoid arthritis in 2012, and evidence has been collected for its beneficial effects on other systemic autoimmune diseases, including systemic lupus erythematosus, systemic sclerosis, polymyositis and large-vessel vasculitis [104,105].

### 4.2. Osteoarthritis 

Differently from rheumatoid arthritis, osteoarthritis (OA) is not considered a wholly acknowledged inflammatory disease, and its etiology, which is not fully characterized yet, encompasses mechanical factors, such as injury, and risk factors, including obesity and age. Nevertheless, synovial macrophages that release proinflammatory cytokines, especially TNF, are acknowledged to be implicated in the pathological chain of events eventually leading to deterioration of cartilage in the joint, which is a hallmark of the disease [106]. High levels of solTNF are associated with OA, implying enhanced activity of ADAM17 in the disease. This, in turn, suppresses synthesis of the major cartilage components, such as proteoglycans and fibrillar type II collagen [107], and promotes the release from chondrocytes of the cartilage degrading proteases MMP-1, MMP-3 and MMP-13 [108,109]. Despite a clear involvement of TNF in the pathophysiology of OA, clinical trials of TNF inhibitors have not yielded conclusive results and they are generally believed to not be beneficial in OA [110].

Aggrecanases and collagenases are the main players in cartilage degradation. Extracellular levels of several of these proteases are regulated by the ADAM17 substrate endocytic receptor low-density lipoprotein receptor-related protein 1 (LRP1) [111], including ADAMTS-1, -4 and -5 and MMP-1 and -13 [112,113,114,115,116]. Yamamoto and colleagues showed that LRP1 inactivation through ADAM17-mediated shedding is a crucial event in the pathophysiology of OA [117]. ADAM17 inhibition restores LRP1 activity and reverts cartilage degradation. Moreover, shedding generates the soluble form of LRP1, sLRP1, which can function as a decoy receptor for cartilage-degrading proteases, further preventing their internalization. LRP1 shedding is associated with inflammatory conditions, and synovial macrophages, in addition to releasing TNF and other pro-inflammatory cytokines, also represent the major source of sLRP1 in OA joints [118]. It is conceivable that inactivation of ADAM17 in synovial macrophages, potentially by targeting iRhom2, would reduce production of TNF and sLRP-1 in OA joints, without affecting other physiological activities of ADAM17 that, in cells such as chondrocytes, could be supported by iRhom1.

### 4.3. Lung Pathology

Arndt and colleagues investigated the role of leukocyte-associated ADAM17 in acute lung inflammation by using a conditional knock-out mouse lacking ADAM17 in hematopoietic cells and their progenitors. Following LPS inhalation, a lack of ADAM17 in leukocytes reduced levels of alveolar TNF and L-selectin, and, as a consequence, promoted a reduction in neutrophil infiltration and lung inflammation [119]. Furthermore, in a similar model of acute lung injury induced by intranasal LPS, a lack of ADAM17 in endothelial cells (Tie2-adam17-/- mice) reduced shedding of the junctional adhesion molecule JAM-A and the transmembrane chemokine CX3CL1, in addition to decreased levels of solTNF and IL-6. As a consequence, these mice had reduced vascular permeability, edema formation and pulmonary leukocyte recruitment [120]. Altogether, this evidence suggested that ADAM17 could be a potential target in the design of pharmacologic therapies for acute lung injury.

In addition, ADAM17 plays a crucial role in activating pathological airway remodeling in lung diseases, including asthma, chronic obstructive pulmonary disease (COPD) and cystic fibrosis (CF). Cues triggering ADAM17 activation can be different, but they lead to a similar EGFR signaling and molecular events characteristic of pathological airway remodeling [121]. Indeed, in both COPD and CF, ADAM17 and the consequent EGFR signaling can be aberrantly activated, thereby leading to airway epithelial cell wound healing, abnormal airway proliferation, maintenance of barrier integrity and progressive lung tissue scarring that are features of both diseases [121,122].

### 4.4. Atherosclerosis

The effect of ADAM17 deficiency on the progression of atherosclerosis was analyzed by crossing the ADAM17 hypomorphic mouse (Adam17^ex/ex^) with the Ldlr^−/−^ mouse, an established model of the disease [123]. Although solTNF plays a negative role in the development of the disease, Nicolau and colleagues found that the Adam17^ex/ex^-Ldlr^−/−^ mice developed larger atherosclerotic lesions than wild-type littermate controls [123,124]. This was a consequence of reduced ADAM17-mediated shedding, which led to increased levels of tmTNF and TNFR2, thereby resulting in a constitutive activation of TNFR2 signaling. In turn, excess of TNFR2 signaling promoted proliferation of macrophages and their augmented recruitment to the lesion sites, where they actively participate in cholesterol accumulation and formation of atherosclerotic plaques [123].

### 4.5. Inflammatory Bowel Disease

Inflammatory bowel disease (IBD) is a wide term that describes disorders of the digestive tract involving chronic inflammation. The two main types of IBD are Crohn’s disease and ulcerative colitis. High levels of TNF and enhanced ADAM17 activity were found in the intestinal mucosa of IBD patients, indicating a crucial role for the protease in the development of the disease [125,126]. In agreement, anti-TNF therapy is effectively used in treatment of IBD [127]. However, in vivo studies indicated a more complex role for ADAM17 in regulating the homeostasis of intestinal barrier in the context of IBD. For example, ADAM17 was found to be protective, rather than detrimental, in the development of ulcerative colitis (UC) by promoting epithelial cell growth and goblet cell differentiation. Mice with systemic deletion of ADAM17 (Adam17^flox/flox^Mx1-Cre knock-in mice, in which temporal systemic deletion of ADAM17 is obtained by injection of plpC–polyinosinic polycytidylic acid) developed more severe dextran sulfate sodium-induced colitis when compared to control littermates [128]. ADAM17 was predominantly expressed by regenerating epithelia in control mice, and its loss diminished EGFR activation and subsequent epithelial proliferation. Conversely, ectopic injections of TGFα restored EGFR signaling and barrier functions [128]. Similarly, the ADAM17^ex/ex^ mouse displayed increased susceptibility to dextran sulfate induced colitis, due to a lack of EGFR signaling and STAT3 activation [16]. In UC patients, epithelial ADAM17 expression positively correlated with both cell proliferation and goblet cell number. Furthermore, a rare loss-of-function mutation in ADAM17 was identified in human. This caused a syndrome characterized by bowel disease, additionally to skin disorders [129]. Interestingly, loss of iRhom2 in mice did not exacerbate dextran sodium-sulfate induced colitis, in agreement with the redundant function of iRhom1 to support shedding of EGFR ligands. Nevertheless, a lack of iRhom2 promoted spontaneous colitis in IL10-deficient mice [130]. Although the intestinal barrier was not compromised by deficiency of iRhom2 in this model, colitis was caused by dysregulated host immune responses to the gut microbiota, providing the first evidence that iRhom2 can regulate the homeostasis between host and microbiome [130].

### 4.6. Neurodegeneration and Alzheimer’s Disease 

Neuroinflammation has emerged as a crucial component of neurodegeneration. While different diseases, including Alzheimer’s, multiple sclerosis and amyloid lateral sclerosis, are initiated by different causes and affect different compartments of the nervous system, microglia are thought to play a similar role in these diseases, integrating pathological stimuli and production of cytokines that ultimately promote neuronal loss [131]. Among these cytokines, TNF plays a key role, especially in Alzheimer’s disease (AD). TNF has a genetic link with AD as TNF polymorphisms, leading to an increased expression of the cytokine are associated with its pathogenesis [132]. The function of ADAM17 in neurodegenerative diseases has not been fully elucidated yet because of a lack of suitable knockout mice; thus, we can predict its role based on in vivo investigations of TNF in neuroinflammation. TNF knock out mice have reduced remyelination in models of multiple sclerosis and increased lesions in cerebral ischemia models, indicating a protective role for the protein [133,134]. On the other hand, ablation of TNF in a mouse model of AD lowered cognitive decline, although it did not affect the deposition of Aβ plaques, a hallmark of the disease [135]. As previously mentioned, TNF receptors can trigger completely opposite cell responses. In agreement, the ablation of TNFR1 or 2 lead to a diverse outcome in neurodegenerative diseases. A lack of TNFR1 in a murine model of AD reduced plaque deposition microglia activation [136]. Similarly, intracerebroventricular infusions of Aβ oligomers, which led to cognitive decline in wild-type mice, failed to trigger cognitive impairment in TNFR1-deficient mice [137]. Given its role in extinguishing TNF pro-inflammatory signaling, TNFR2 is considered to be protective in neurodegenerative diseases. In agreement, TNFR2 ablation impairs cognition in mouse [138]. Interestingly, ablation of both TNF receptors accelerated AD [139]. Nevertheless, anti-TNF inhibitors, which block both TNFR1 and 2 signaling pathways, have shown positive results in ameliorating the pathology in murine models of AD and AD patients. Infliximab reduced plaque deposition and microglia activation in a mouse model of the disease [140]. Intrathetical injection of infliximab improved AD in a Chinese woman [140]. Finally, an engineered TNF inhibitor that is selective for solTNF over tmTNF lowered plaque deposition when administered to a mouse prone to develop the AD pathology [141].

In addition to contributing to AD by controlling the solTNF/tmTNF switch in microglia, ADAM17 is thought to be involved in the pathogenesis of the disease by regulating the shedding of the amyloid precursor protein (APP), the protein whose cleavage is known to trigger the amyloid cascade leading to AD [142]. While ADAM10 has been reported to be the physiological “constitutive alpha-secretase” of APP, ADAM17 is known to be the “stimulated alpha-secretase” [143,144]. Both ADAM10 and ADAM17 are generally considered beneficial in AD, as they promote the anti-amyloidogenic processing of APP that counteracts its amyloidogenic release and generation of the pathogenic Aβ peptide. Although ADAM17′s protective role in the development of AD is mainly based on in vitro studies and in vivo studies are currently missing, in support of this evidence it has been recently reported that a rare variant leading to loss-of-function of ADAM17 is associated with the pathogenesis of AD in human [145]. Given its pivotal role in orchestrating TNF responses and APP shedding, it is easy to speculate that ADAM17 may promote a dual and opposite effect on development of the disease. Neuron-associated ADAM17 may have a beneficial effect by triggering the non-amyloidogenic pathway of APP processing, while microglia-associated ADAM17 can be detrimental for its ability to release TNF and sustain chronic inflammatory responses. On these premises, it is clear why iRhoms represent a promising therapeutic target in AD. As a consequence of their peculiar tissue expression, with only iRhom1 expressed in neurons, but not iRhom2, and only iRhom2 expressed in microglia, but not iRhom1, the ability of ADAM17 to process TNF or APP can be differentially regulated by either iRhom [146]. Thus, a potential inhibition of iRhom2 would inactivate ADAM17 in microglia, thereby preventing the pathological cleavage of TNF, but not in neurons, where iRhom1 would still support the ADAM17-dependent non-amyloidogenic process of APP and all the other physiological functions of the protease in the brain. In agreement with its role in promoting TNF release and neuroinflammation, iRhom2 has been identified as a genetic risk factor in AD [147].

### 4.7. Nerve Recovery 

ADAM17 plays a negative role in nerve regeneration. Both the ADAM17 hypomorphic mouse (ADAM17^Ex/Ex^) and a mouse model with deletion of ADAM17 in microglia displayed a better functional recovery after spinal cord injury (SCI) [148]. This phenotype, which was not reproduced by deletion of ADAM17 in endothelial cells or macrophages, was characterized by higher clearance of apoptotic cells and augmented axon growth, potentially due to higher levels of the phagocytic receptor CD36, which is upregulated in ADAM17-deficient mice. Another possible mechanism explaining the increased phagocytic capacity of ADAM17-deficient microglia can be linked to a lack of TREM2 shedding. TREM2, which has been found to be an ADAM17 substrate, plays a central role in microglia activation and its capability to clear debris [149,150].

### 4.8. ADAM17 in Cancer

Given its ability to shed pro-inflammatory molecules, pro-tumorigenic substrates, adhesion molecules and other molecules involved in cancer progression, ADAM17 plays a multifunctional role in cancer progression that can vary among different tumors and stages of the disease. As a consequence of its capability to trigger the epidermal growth factor receptor (EGFR) pathway by shedding EGFR ligands, ADAM17 activity is associated with cancer progression of several malignancies, including colon and breast cancer [10,151,152]. Ablation of ADAM17 protects against the progression of colon cancer in an in vivo model of the disease due to reduced shedding of amphiregulin and activation of EGFR signaling [153]. Similarly, inhibition of ADAM17 reduced growth of xenografts of human colorectal adenocarcinoma and mouse colon carcinoma cells in vivo [154,155]. ADAM17 promotes progression of breast cancer by regulating levels of TGFα, which plays a pivotal role in this pathological process [156,157]. Inhibition of ADAM17 and TGFα shedding reduced proliferation of triple-negative breast cancer cells in vitro [158]. Furthermore, high levels of soluble EGFR ligands and elevated expression of ADAM17 correlate with poor cancer prognosis. In addition to its extensively investigated role in cancer progression through modulation of the EGFR signaling pathway, ADAM17 regulates other features that are associated with the disease, which have been more recently characterized. For example, ADAM17 is activated by VEGF-A and plays a crucial role in pathological neovascularization, a key feature of cancer [159]. Ablation of ADAM17 in endothelial cells decreased neovascularization in vivo. This phenotype could be largely restored by addition of the ADAM17 substrate HB-EGF, indicating that ADAM17 elicits this function by mediating the crosstalk between the angiogenic receptor VEGFR-2 (or FGFR2) and EGFR.

ADAM17 activity is also associated with immune evasion of cancer cells by modulating the cleavage of programmed death-ligand 1 (PD-L1) [160]. PD-L1 is a type one transmembrane protein, expressed at high levels in several human cancers and playing a major role in suppressing adaptive immune responses [161]. PD-L1 transmits inhibitory signals to CD8+ T cells and CD4+ helper cells through its cognate receptor PD-1. As a consequence, clonal expansion of antigen-specific T cells is reduced and their immunosurveillance against cancer cells attenuated [162]. ADAM17 proteolytically processes PD-L1 and releases its ectodomain in the extracellular milieu [160]. High levels of ADAM17 and soluble PD-L1 are associated with poor cancer prognosis. Furthermore, this process has high relevance in the treatment of cancer as increased soluble PD-L1 promotes resistance to PD-L1 inhibitors. Indeed, soluble PD-L1 induces apoptosis of CD8+ T cells, thereby compromising their ability to kill tumors [163]. Additionally, ADAM17 promotes immune evasion by shedding of CD16A, a human IgG Fc receptor specifically expressed on NK cells, which is able to bind the constant fragment of antibodies, thereby triggering NK cell activation and antibody dependent cell mediated cytotoxicity (ADCC). ADAM17-dependent cleavage of CD16A promotes reduction of CD16A on the surface of NK cells, and subsequent dampening of its signaling and cytokine production [163,164]. In agreement, genetical ablation of ADAM17 on circulating NK cells by CRISPR/CAS9 led to their improved activity, with augmented cytokine production and cancer cell cytotoxicity compared to wild type controls [162].

Taken together, these results indicate a multifunctional role for ADAM17 in cancer. Generally, high levels of ADAM17 are associated with poor cancer prognosis due to the exacerbation of EGFR signaling, but it can play additional roles in different phases of cancer progression. Moreover, novel evidence has emerged that ADAM17 can modulate the capability of cancer cells to evade immunosurveillance and driving cancer evasion, by regulating cleavage of crucial immune receptors and ligands in both immune and target cancer cells [161,162].

### 4.9. COVID-19

The angiotensin converting enzyme 2 (ACE2) is the entry receptor for the severe acute respiratory syndrome coronavirus 2 (SARS-CoV-2), the virus causing the coronavirus disease-19 (COVID-19) [165]. The virus entry is known to activate ADAM17 [166]. In addition, ADAM17 is a major ACE2 sheddase, and, as such, it regulates the virus entry and it is clearly involved in the pathophysiology of the disease [167]. Recombinant soluble human ACE2, which mimics the activity of an ACE2 shed variant, prevents the virus from entry and diminishes infection in human organoids(Table 2) [168]. However, despite this evidence indicating a beneficial role for ADAM17 in the pathophysiology of the disease, other investigations have suggested a detrimental role for the protease in development of COVID-19 [169]. High levels of ACE2 in the plasma, which may be a consequence of enhanced ADAM17 activity, correlate with severity of COVID-19; higher levels of ACE2 can be found in the plasma of smokers, individuals affected by diabetes, chronic obstructive pulmonary disease and other conditions that are considered COVID-19 comorbidities [170]. The detrimental consequences of ADAM17 activity in COVID-19 may be linked to TNF release and its contribution to the “cytokine storm,” a feature of the disease that leads to vascular hyperpermeability, multiorgan failure and death [171]. Moreover, ADAM17-mediated processing of ACE2 in response to the virus can decrease cellular levels of functional receptor, thereby deregulating the renin-angiotensin system with detrimental effects on the outcome of COVID-19 patients [172]. In conclusion, it is clear that ADAM17 plays a crucial and multifactorial role in the development of COVID-19. However, whether effects of the protease activity are beneficial or detrimental in the disease is unclear as of yet, and additional in vivo studies would be required to address this question.

## 5. Strategies for ADAM17 Inhibition

### 5.1. Small Molecule Inhibitors

**Figure 3:**
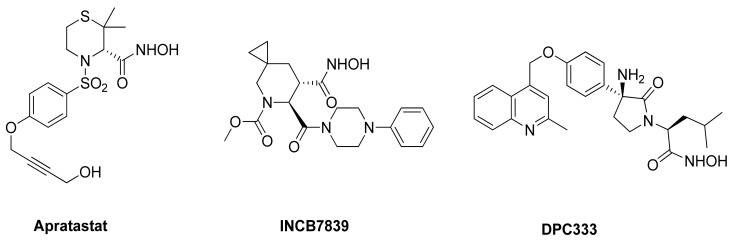
Chemical structure of ADAM17 inhibitors entered in clinical trials.

As mentioned in the previous paragraphs, ADAM17 is involved in ectodomain shedding of cell surface molecules, regulating different pathological and physiological cellular processes. The selective inhibition of ADAM17 would allow to avoid side effects due to the blockade of physiological pathways, but the high homology among metzincin catalytic domains have hampered the discovery of selective inhibitors. Among others, ADAM10 is the closest homologue of ADAM17, sharing several structural and functional characteristics [173]. Thus far, many efforts have been directed to develop small molecules as selective ADAM17 inhibitors, but none of these compounds is available on the market as a drug. Several compounds were recently entered into clinical trials, but they were subsequently withdrawn. Apratastat (Wyeth pharmaceuticals), DPC 333 (Bristol-Myers Squibb Company) and INCB7839 (Incyte corporation) (Figure 3) are the most studied ADAM17 inhibitors, although they failed in phase-II of clinical trials owing to their toxicity [174,175]. In detail, Apratastat, in addition to a lack of efficacy, was terminated for adverse events emerged in 7 out of 390 RA patients. DPC 333 was terminated because of a lack of efficacy in treatment of RA and liver toxicity induced by mechanism-based inhibition of ADAM17.

Finally, INCB7839 was tested in HER2^+^ breast cancer phase I/II trial, but its development was terminated due to contradictory results.

**Figure 4:**
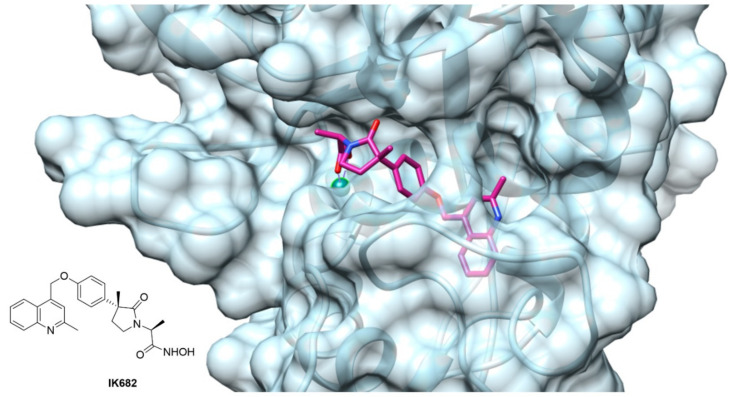
ADAM17 catalytic binding site complexed with compound IK682, a DPC-333 analogue [179] (PDB: 2FVS. Image created using Chimera, version 1.13).

The crystallographic studies of ADAM17 structure revealed a particular ellipsoid shape of ADAM17 catalytic domain. In fact, it is possible to identify two subdomains separated by an active site cleft where the catalytic zinc ion is arranged (Figure 4) [176]. Here, the cleavage site separates the “primed” *C*-terminal side, from the “unprimed” *N*-terminal side. In the unprimed side are allocated the classical S1, S2 and S3 pocket of the enzyme, corresponding to the P1, P2 and P3 of the substrate, while the pockets on the primed side of the zinc are called S1′, S2′ and S3′. ADAM17 presents the unique feature of a “L-shaped” S1′ pocket. This pocket forms a deep channel close to Leu384 and Ala439 with the S3′ pocket, creating a polar entrance between S1′ and S3′.

The X-ray structure of the ADAM10 ectodomain has been recently solved and revealed a high similarity with the ADAM17 catalytic domain [177]. They slightly differ only in their S1′ pocket that in ADAM10 is a deep hydrophobic canyon with specific residues (V376, I379, T380, I416 and T422) able to interact with bulky hydrophobic substrates, including aromatic and polyaromatic functions. In contrast, ADAM17 S1′ pocket is shallower and constrained by Ala439 and Val440, thus preferring smaller hydrophobic residues [178].

The majority of the small molecule ADAM17 inhibitors reported so far contain a peptido-like backbone allocated in the “primed” side, a lipophilic substituent fitting the S1′ specificity pocket and a zinc-binding group (ZBG). The most common structure comprises a sulfonamide or amide group as hydrogen bond acceptor, an aromatic or polyaromatic group interacting with the S1′ pocket and a hydroxamic acid as ZBG. For an ADAM17 inhibitor to be selective over the other ADAMs and MMPs, thus avoiding the off-target side effects, it must have a functional interaction with the “L-shaped” S1′ pocket. Figure 4 shows the crystal structure of ADAM17 (light blue) complexed with IK682 (magenta), an analogue of the most studied inhibitor DPC-333 [179]. The phenoxy group of IK682 perfectly interacts with the S1′ pocket, while the methyl quinoline portion is allocated to the channel between the S1′ and S3′ pockets.

The majority of small molecule inhibitors of ADAM17 reported in the last 10 years have been extensively reviewed in several articles [10,180,181,182]. For this reason, in the present review we focus our attention on ADAM17 synthetic inhibitors published since 2015. Novel compounds have been classified on the basis of their chemical structure and mechanism of interaction with the enzyme in: hydroxamate-based, non-hydroxamate-based and non-zinc-binding inhibitors.

#### 5.1.1. Hydroxamate-Based ADAM17 Inhibitors

Inspired by the Apratastat (TMI-005) and DPC-333 structure, Ouvry et al. carried out a survey of the literature to understand which are the fundamental groups of these two potent inhibitors to be exploited for the synthesis of new ADAM17 inhibitors for a topical application [183]. Apratastat and DPC-333 have been used as lead compounds, and the insertion of novel cyclic linkers attached to hydroxamate was explored to develop a new class of inhibitors. Among them, compound **1** (Table 3), featuring a methyl quinoline group in P1′ and a piperazine ring as a linker for the ZBG, was identified. It showed nanomolar activity for the target enzyme and was selective over the other tested ADAMs and MMPs. Unfortunately, the weak potency revealed in cell assay on keratinocytes (Table 4) precluded further investigations of this compound.

In order to avoid the enzyme-cell drop-off of activity experimented with compound **1**, the same group adopted an assay based on TNF inhibition in human peripheral blood mononuclear cells (PBMC) to directly evaluate activity of a new series of sulfonamide-based hydroxamate derivatives [184]. Among them, the quinoline derivative **2** (Table 3) with a *N*-acetylated azetidine linker was the most promising inhibitor with excellent enzymatic inhibitory activity (IC_50_ = 4 nM) and selectivity profile, also confirmed in an oxazolone-induced chronic skin inflammation model in mice (Table 4). In fact, it was selected as a clinical candidate for the topical treatment of psoriasis.

An innovative reverse hydroxamate-based ADAM17 inhibitor KP-457 (compound **3**, Table 3) was recently reported by Hirata et al. [185]. KP-457 showed a nanomolar activity for the target enzyme and a high selectivity over ADAM10 and MMPs. By selectively blocking ADAM17, KP-457 was able to preserve the activity of the glycoprotein Ibα (GPIbα), the von Willebrand factor receptor, on the surface of human induced pluripotent stem cells (iPSCs) that are used to produce in vitro functionally active platelets for transfusion.

#### 5.1.2. Non-Hydroxamate-based ADAM17 Inhibitors

It is well known that the presence of a strong ZBG, such as a hydroxamate, can cause side effects and toxicity due to off-target inhibition of other metzincins [196]. For this reason, the research of new ADAM17 inhibitors has been directed to use “soft” ZBGs as an alternative to hydroxamic acid. Hydantoin-based ADAM17 inhibitors were extensively studied by Merck. In 2010, a promising acetylene-based hydantoin derivative was reported by Girijavallabhan et al. (compound **4**, Table 3) and was the starting point to explore hydantoin-derived ADAM17 inhibitors with improved bioavailability and better pharmacokinetic profile [186]. In 2017, Tong et al. modified the hydantoin-based structure by replacing the pendant acetylene with an aza benzofuran group. The insertion of this new moiety conferred high potency in a human whole blood assay (hWBA) and a good pharmacokinetic profile to the new derivatives [187]. Moreover, polar functionalities, especially basic groups and H-bonding donor groups, were inserted in the benzofuran ring reporting good results in term of potency. The aza benzofuran hydantoin **5** (Table 3) was identified as the lead compound and was chosen for further investigations. In fact, in the following paper by the same group, the structure of derivative **5** was modified to improve the oral absorption and the membrane permeation [188]. Different removable substituents were inserted on the keto amide NH of hydantoin ring. The best results were displayed by the pivalate prodrug **6** (Table 3), presenting a rapid rat AUC of 13.1 μM.h and appreciable DMPK properties evaluated through oral administration in fasted rats, dogs and monkeys. Therefore, compound **6** was selected for further preclinical evaluation.

A thiadiazolone derivative JTP-96193 (**7**, Table 3) was recently reported by Japan Tobacco, Inc. [189]. This new compound presented a nanomolar activity for ADAM17 and an excellent selectivity profile (with a >1850-fold selectivity over ADAM10). Moreover, it was pharmacologically tested on type 2 diabetes and diabetic peripheral neuropathy (DPN) in mouse model of obesity and diabetes. JTP-96193 reduced the TNF release from fat tissue preventing diabetes development and improving the insulin resistance. Furthermore, the administration of JTP-96193 prevented the development of DPN in streptozotocin (STZ)-induced diabetic mice, without any effect on glucose blood level and insulin resistance (Table 4).

In 2015, Leung et al. reported the first metal-based inhibitor of ADAM17, the iridium(III)-based complex **8** (Table 3) [190]. The authors screened an in-house library of structurally different complexes, highlighting the importance of the charge localization within the metal complex for activity. Compound **8** revealed a µM ADAM17 inhibitory activity evaluated by a fluorometric assay on recombinant enzyme, without any data regarding the selectivity profile. Complex **8** was tested on human monocyte THP-1 cell line stimulated with LPS (Table 4), and it was able to inhibit TNF secretion and p-38 MAP kinase phosphorylation. This complex was considered a lead compound deserving further optimization to develop more effective ADAM17 inhibitors.

A molecular docking study on ADAM17 in order to design new non-hydroxamate sulfonamide ADAM17 inhibitors was reported by Sarkate et al. [191]. The P1′ and the zinc binding sites have been analyzed by docking studies through rigid protein-flexible ligand docking and flexible ligand-flexible protein docking. The model revealed a similar binding pose in the S1′ pocket for three different aromatic moieties (isoquinoline, naphthalene and quinoline) and a good fitting inserting a carboxylate group as ZBG. Moreover, a longer side chain conjugated to the α position of carboxyl group improved the selectivity of sulfonamides toward ADAM17 over MMPs. The synthesis and preliminary activity of some promising carboxylate sulfonamides has been reported. Compound **9** (Table 3) revealed the best ADAM17 inhibitory activity (68.48% inhibition of TNF concentration) using a rat TNF ELISA kit. Moreover, the in vivo anti-inflammatory activity was evaluated by carrageenan-induced rat paw edema model, and sulfonamide **9** showed a good anti-inflammatory activity (about 60% of inflammation inhibition) (Table 4).

#### 5.1.3. Non-Zinc-Binding ADAM17 Inhibitors

A recent approach pursued to overcome cross-inhibition of other zinc-containing metalloproteinases consists of designing compounds interacting with ADAM17 outside the catalytic binding site. In 2012, Minond et al. described the first non-zinc binding ADAM17 inhibitor (compound **10**, Table 3), a piperazine-2,3-dione derivative, discovered through high-throughput screening assays using glycosylated and non-glycosylated substrates [192]. The adopted strategy was to use the ADAM protease ability to accommodate glycosylated substrates in a secondary binding site (exosite). Later on, the same group reported a structure-activity relationship (SAR) study of this class of exosite-targeting inhibitors, and the most promising candidates were also analyzed by biochemical and cell-based assays [193]. The data confirmed compound **10** (Table 4) as the most promising ADAM17 inhibitor of this series, being able to selectively inhibit ADAM17 in cell-based assays and demonstrating an unusual substrate selectivity by sparing ADAM17-mediated cleavage of TGFα.

Even though compound **10** did not suppress the shedding of some cell-surface proteins (such as FKN and CXCL16 in A549 cell line and TGFα in A549), it was able to prevent the shedding of different substrates such as EGFR ligands (heregulin in A549 cells), receptors (discoidin receptor 1 (DDR1) in HCC1806 breast cancer cells and protein kinase 7 (PTK7) in human fibrosarcoma cell lines HT1080 and L622D) and cytokines (TNF in THP1 cells and IL-8 in human tracheal smooth muscle cell HTSMC) in different cell models. This substrate-selective inhibition, never reported before, was hypothesized to be due to the interaction of compound **10** with just one exosite of ADAM17 responsible for the binding of specific substrates.

In 2018, a promising non-zinc-binding inhibitor, the thioxodihydro pyrimidindione ZLDI-8 (**11**, Table 3), was discovered by performing a pharmacophore-based virtual screening on SPECS compound database [195]. ZLDI-8 in combination with 5-fluorouracil or irinotecan synergistically decreased the anti-proliferative and anti-metastatic effect on colon rectal cancer (CRC) cells by reversing Notch and epithelial-mesenchymal transition (EMT) pathways. ZLDI-8 sensitized CRC cells to the activity of these anti-tumoral drugs by acting as a new adjuvant agent.

The same group reported the activity of ZLDI-8 as an ADAM17-specific inhibitor able to disrupt the Notch pathway in hepatocellular carcinoma (HCC) cells, avoiding the NICD (Intracellular domain of Notch) accumulation in the nucleus, and inhibiting the EMT process of HCC cells [194]. The inhibition of Notch pathway using this new ADAM17 inhibitor decreased the expression of anti-apoptosis, prosurvival and EMT related genes. Furthermore, an improved susceptibility of HCC cells to Sorafenib, Etoposide and paclitaxel after ZLDI-8 administration was observed. In particular, an enhanced effect of Sorafenib on inhibiting HCC is evidenced in vivo (Table 4).

In a further study, ZLDI-8 was shown to inhibit migration and invasion in a highly aggressive type of HCC cells (MHCC97-H and LM3) in vitro and to block lung metastasis in vivo [197]. In 2019, ZLDI-8 was also tested on chemo-resistant non-small cell lung cancer (NSCLC) [198]. This Notch-signaling inhibitor was able to induce apoptosis in lung cancer cells, also reducing migration, invasion and EMT phenotype of drug-resistant lung cancer cells. In fact, ZLDI-8-induced apoptosis of A549 and A549-Taxol cells through the mitochondrial signaling pathway in vitro and suppressed the growth of a multidrug-resistant lung cancer xenograft in vivo. Moreover, a decrease in metastasis development was achieved by its administration in a tail vein injection mice model.

### 5.2. Pro-Domain–Dominant Negative Forms of TACE

Specific ADAM17 inhibition through synthetic compounds has been proven to be challenging due to the highly conserved catalytic domain of different metalloproteases. Thus, there has been growing interest in developing ADAM17 inhibitory biomolecules based on the biology of the enzyme and its physiological mechanisms of inhibition. One of the most investigated mechanisms for its therapeutical potential is the auto-inhibition of ADAM17 by its prodomain (TACE pro-domain or TPD). This strategy is based on the evolutionary function of TDP, which is essential for a correct folding of ADAM17 and ensures its inactivation throughout the secretory pathway, thus preventing unspecific cleavage of ER-associated proteins. In contrast to the high similarity of the catalytic domain among different metalloproteases, TDP has only 23% similarity with the prodomain of ADAM10, ADAM17 closest relative within the ADAM family. Despite its potential, developing an efficient ADAM17 TDP to be used in therapy has been challenging for a number of reasons. First, TDP functions as a chaperone for the catalytic domain of ADAM17, and it has limited access when the catalytic domain is already folded. Thus, TDP has a relatively low K_i_ for ADAM17 when it is used in trans, majorly due to the cysteine rich and disintegrin domain that structurally hinder the access of TDP to the active site when ADAM17 is already folded. Secondly, TDP does not fold correctly when expressed in *E. coli*. Aided refolding of TPD inclusion bodies results in altered binding properties of the inhibitor against ADAM17. Thus, production of functional TDP has been complicated and only recently, due to improved biotechnology techniques, use of TPD in ADAM17-associated diseases has received growing interest [199]. ADAM17 TDP has shown to efficiently inhibit ADAM17 functions in vivo. ADAM17, via shedding of TGFα and activation of EGFR, plays a negative role in neuronal regeneration after brain traumatic injury, by favoring gliogenesis [200]. Local injections of lentiviral-based TPD constructs at the site of injury promoted blockage of the ADAM17/TGFα/EGFR signaling cascade, decreased deposition of gliogenic environment, thus promoting neurite outgrowth and brain repair [200]. In addition, administration of recombinant TPD ameliorated the pathology in two well-characterized murine models of inflammatory diseases, the 2,4,6-Trinitrobenzenesulfonic acid (TNBS)-induced colitis and collagen-induced arthritis [201]. In addition, Soto-Gamez and colleagues have developed an innovative therapeutic approach to target the function of ADAM17 in EGFR activation and cancer progression. They fused TDP with a competitive not-active EGFR-binding protein called DARPinE01. The resulting biomolecule had greater effects than non-engineered TPD on reducing migration and invasion of cancer cells [202].

### 5.3. Substrates Glycosylation

Analysis of ADAM substrates revealed that glycosylation is often present in the vicinity of the scissile bond, and this post-translational modification enhances their susceptibility to ADAM17 by forming kinetic interactions with exosites of the enzyme. A large compound library was screened in order to identify molecules that differentially inhibit the cleavage of glycosylated or non-glycosylated substrates of ADAM17 [192]. Inhibitors identified with this strategy were directed against exosites of the enzyme, rather than against the conserved metalloprotease catalytic domain, and therefore, resulted in very selective compounds for ADAM17 over other metalloproteinases, such as ADAM10.

### 5.4. Dominant Negative Forms of ADAM17

Solomon and colleagues have developed a strategy for ADAM17 inhibition based on a deletion mutant of the enzyme, lacking the catalytic domain [203]. This protein acts as a dominant negative form of ADAM17 and was proven to block TNF release and TNFR2 shedding. A similar approach was used to inhibit the activity of proteases that need to dimerize in order to cleave their substrates, as occurs for MT1-MMP [204]. However, at the time of the discovery, it had not been reported that ADAM17 could dimerize at the cell surface, and the authors suggested that the dominant negative form of ADAM17 could somehow interfere with the function of the endogenous proteinase, either by binding and sequestering TACE substrates via the ancillary domains or by some other mechanism that had yet to be determined. Although dimerization of ADAM17 at the cell surface had been later reported in one isolated study, the inhibitory mechanism of the dominant negative form of ADAM17 has become clearer after iRhoms were characterized [205]. Indeed, given that the interaction between ADAM17 and iRhoms is essential for the protease function, it is easy to speculate that the dominant negative form of ADAM17 can compete with endogenous ADAM17 for its binding to iRhoms, thereby inhibiting the protease maturation and activity [61].

### 5.5. Engineered TIMPs

Among the four mammalian TIMPs, TIMP-3 is unique in its ability to inhibit ADAM17. Before that the crystal structure of ADAM17/TIMP-3 complex was resolved, Gillian Murphy’s group had already identified determinants of the ADAM17 inhibition by TIMP-3 and developed engineered TIMPs with high specificity for the proteinase [206]. Based on computer models of molecular docking, sequence alignment between TIMPs and systematic introduction of point mutations, the group identified a number of specific residues that play pivotal roles in the interaction between TIMP-3 and ADAM17. First, they demonstrated that mutating Ser4, a residue with a short side chain, with other residues that better fulfill the highly hydrophobic and deep S1′ pocket of the enzyme (~5.5–6.0 Å), renders TIMP-3 a better ADAM17 inhibitor [207]. Then, they found that the AB loop of TIMP-3 plays a pivotal role in ADAM17 inhibition. Introduction of the AB loop of TIMP-3 into TIMP-1 as a scaffold, together with specific TIMP-3 residues that were thought to take critical contacts with the protease (Ser4; Leu67 and Arg84), makes TIMP-1 able to inhibit ADAM17 [208]. These studies laid the foundations for the development of engineered TIMP-3 proteins that were able to inhibit ADAM17, but not MMPs, and that could be used in the therapy of arthritis. As mentioned before, Cys1 of TIMPs plays a key role in the coordination of the catalytic zinc of metalloproteinases, and the residue in the second position, which is a threonine in TIMP-3, interacts with the S1′ pocket of the enzyme. Wei and colleagues inserted mutations in the TIMP-3 sequence in order to perturbate the interaction between these crucial residues and their residue partners in the active site cleft of the enzyme, thereby disrupting the inhibitory activity of TIMP-3 against MMPs [208]. Thus, they inserted an N-terminal alanine extension (-1Ala) to perturb the interaction of Cys1 with the Zn^2+^ in the active site, and a threonine to glycine mutation in position 2 (T2G), which disrupts the interaction of TIMP-3 with the S1′ specificity pocket of MMPs. Interestingly, both -1Ala-TIMP-3 and T2G-TIMP-3 lost their inhibitory capability against MMPs, but retained it against ADAM17. These two mutants efficiently blocked TNF release in PMA stimulated macrophage-like cells. In addition, -1Ala-TIMP-3 and T2G-TIMP-3 were able to inhibit the aggrecanases (ADAMTS-4 and -5) and block aggrecan degradation, which is, together with TNF release, a key feature of arthritis [209]. Ultimately, the overexpression of -1Ala-TIMP-3 ameliorates the pathology in a mouse model of osteoarthritis, not only compared to wild-type controls, but also to TIMP-3 overexpressing mice, suggesting that a specific inhibition of TACE and aggrecanases over other metalloproteases is highly desirable for development of therapies [210].

### 5.6. Antibodies

Phage display technology has been extensively used to produce therapeutical molecules, including inhibitory antibodies that are able to block the activity of specific proteases [211]. Given the similarity between the catalytic domain of ADAM17 and that of other metalloproteinase, which have rendered difficult the generation of highly selective therapeutical molecules, phage display technology was used to generate a selective inhibitor of ADAM17. Tape et al. targeted the ancillary domains of ADAM17, which are not present in the matrix metalloproteinases and less conserved than the catalytic domain among ADAMs [212]. They used two rounds of screening of a phage display library: within the first screening, all antibody variable heavy (VH) domains able to specifically bind the ADAM17 disintegrin domain were selected. The second screening was performed against the catalytic active site with a library of variable light (VL) chain. This approach allowed the isolation of an ADAM17 cross-domain inhibitory antibody, called D1(A12), which is a fivefold better inhibitor than TIMP-3 in blocking ADAM17-dependent shedding of TNF and EGF-like ligands. In addition, this cross-domain inhibitory antibody was proven to block tumor growth in vivo, when the human ovarian cancer cell line IGROV1-Luc was xenografted in Balb/c nude mice [213]. By using phage-display technology, Rios-Doria and colleagues developed another ADAM17 inhibitory antibody, MEDI3622, that blocked ADAM17 activity, and consequently, release of TNF in LPS-stimulated mice and tumor progression in a head and neck patient-derived xenograft model [214]. Differently from the cross-domain inhibitory antibody, MEDI3622 was selected by and directly targets the ADAM17 catalytic domain. Authors showed that MEDI3622 was capable to eradicate EGFR/HER dependent tumors in the low nanomolar range.

### 5.7. Targeting iRhom2: Perspectives of a Revolution

ADAM17 has been a major drug target since its discovery, as has been extensively discussed within this review. Nevertheless, development of such therapeutic molecules has been much harder than expected, majorly due to difficulties in accomplishing 1) an ADAM17 selective inhibition over other metalloproteases and 2) its specific inhibition in immune cells, but not other tissues, which would impede TNF release without affecting all other physiological functions of the protease. iRhoms are able to discriminate between ADAM17 and its closest relative ADAM10 [61,64]. Thus, their inactivation affects ADAM17 function but not that other metalloproteases. In addition to their selectivity, the peculiar tissue expression of iRhom1 and 2, with both iRhoms expressed in all tissues and only iRhom2 expressed in immune cells, offers an unprecedented therapeutic opportunity to target ADAM17 function in immune cells, with no risk to impair it in other tissues [65,66]. Indeed, while iRhom2 inactivation blocks ADAM17 activity and subsequent TNF release, systemic functions of the protease are preserved by the redundant activity of iRhom1. In agreement, iRhom2-deficient mice show no evident abnormalities, but they are protected against sepsis, RA, lupus nephritis and hemophilic arthropathy, majorly due to blocking the iRhom2/ADAM17/TNF pathway [63,95,130,215,216]. Furthermore, an iRhom2-targeted therapy would also potentially be preferential to an anti-TNF therapy for a number of reasons. Firstly, as has been discussed within this review, solTNF and tmTNF trigger opposite immune responses by activating a pro-inflammatory pathway through TNFR1 or an anti-inflammatory pathway through TNFR2, respectively. Anti-TNF inhibitors prevent the binding of solTNF to TNFR1, but also the interaction between tmTNF and TNFR2. Inactivation of iRhom2, would push the balance of soluble versus transmembrane TNF towards the latter, thereby supporting immunosuppression rather than inflammation. Secondly, in addition to release of TNF, specific inactivation of ADAM17 in macrophages would also prevent IL-6R shedding and the related pathway, which is crucial in chronic inflammatory diseases. Moving forward, a number of pathological conditions are characterized by enhanced ADAM17-mediated shedding of proteins other than TNF. This is the case of OA, in which ADAM17 mediates the aberrant shedding of LRP1, a key protein that governs extracellular levels of the cartilage-degrading enzymes [117], or AD, in which ADAM17 may contribute to development of the disease by shedding LRP1 and TREM2, two proteins involved in the cellular disposal of Aβ peptide and amyloid plaques [111,150]. For these diseases, the inactivation of iRhom2 could have the dual effect of switching off the initial cause triggering the disease and the inflammatory component that is associated with the disease which guides its development.

## 6. Conclusions

Since 1992, when it was identified as the enzyme responsible for the release of soluble TNF, ADAM17 has been considered as a major research focus and a promising drug target for the therapy of chronic inflammatory diseases. However, therapeutic inhibition of ADAM17 has been historically complicated due to two major reasons: its multifunctionality and its high similarity with other metalloproteases. ADAM17 is ubiquitously expressed in human, and it has been identified as the sheddase responsible for cleavage of over 80 substrates other than TNF. Thus, its systemic inhibition, other than blocking TNF release, ends up in mechanism-side effects for deregulation of physiological processes in diverse tissues from immune cells. In addition, its catalytic domain is highly conserved not only among ADAMs, but also among other metalloproteases of the related families of MMPs and ADAMTs. Inhibitors developed to block ADAM17 activity resulted in cross-reacting with other metalloproteases, thus deregulating physiological processes in which these enzymes play a role and leading to off-target side-effects. To overcome these limitations, several approaches have been utilized to develop molecules able to discriminate between ADAM17 and its relatives, and to inhibit ADAM17 in a specific tissue or cell-type. This review encompassed all these methods, with a major focus on those recently developed, which span from the chemical synthesis of highly specific molecules to engineering of endogenous inhibitors of ADAM17. Intriguingly, what researchers have been struggling to accomplish already exists in nature. In this regard, the discovery of iRhoms showed a revolutionary, yet physiological, way to selectively inhibit ADAM17 in immune cells and over other proteases of the same family. This regulatory mechanism offers an unprecedented opportunity to develop ADAM17-targeting therapeutics, especially in the context of chronic inflammatory diseases that are characterized by a deregulated release of TNF.

## Figures and Tables

**Table 1:** List of known ADAM17 substrates.

Cytokine	Cell-to-Cell Communication	Signaling- Receptors	Cell Adhesion	Cellular Transport	Enzyme	Others
TNFα	Amphiregulin	Axl	ALCAM	SCRB1	ACE-2	APP
CSF-1	HB-EGF	CD16	CD44	LRP-1	Carbonic Hydrolase 9	APLP-2
KL-1	TGFα	CD163	L-selectin	LDL-R	Klotho	Prion protein
KL-2	Epigen	CD30 (TNFRSF8)	Collagen XVII	SORCS-1	NPR1	Vasorin
Lymphotoxin α	Epiregulin	CD40 (TNFRSF5)	Desmoglein-2	SORCS-3		PMEL-17
RANKL	NRG-1	CD89	EpCam	SORL-1		Sydecan-1
Cx3cl1	Jagged	EPCR	GP-1ba	SORT-1		Sydecan-4
IL-8	DLL-1	ErbB-4	GP-5	TREM-2		Pre-adypocyte factor
	PD-L1	GHRH receptor	GP-6	IGF-2R		Collagen IV *
	ICOS-L	M-CSFR	ICAM-1			PCPE-1 *
	IL-15R	Notch-1	JAM-A			cystatin C *
	IL-1R2	NRP-1	L1-CAM			Ebola virus Glycoprotein **
	IL-6R	PTK7	LYPD3			
	LAG-3	PTPRZ	MUC-1			
	MIC-A	PTPRF	NCAM			
	MIC-B	SEMA-4D	Nectin-4			
	TIM-1	TNF-R1	SynCAM-1			
	TIM-3	TNF-R2	VACM-1			
	TIM-4	NTRK1	Thrombospondin-4			
		VEGF-R2				

* Secreted proteins identified as substrates of soluble ADAM17; ** A virus-encoded protein that is released by ADAM17 after viral infection.

**Table 2:** Diseases in which ADAM17 has been implicated.

Pathology	Role	Effects of ADAM17 Inactivation	Substrates	Reference
**Rheumatoid Arthritis**	**Detrimental in the progression of the** **disease**	Decreased inflammation and cartilage breakdown	TNF IL-6R	[95,103]

	Regulates the release of TNF and activation of TNFR signaling			

	Regulates the activation of the pro-inflammatory IL-6 trans-signaling			
**Osteoarthritis**	**Predicted negative role in the progression of osteoarthritis**	Enhanced secretion of metalloproteases and lowered deposition of ECM	TNF LRP-1	[108,109,117]

	Regulates TNF signaling			
	
	Controls levels of the endocytic receptor LRP-1	Decreased metalloprotease turnover		
**Lung** **pathology**	**Negative role in the disease**	Lower neutrophil recruitment and inflammation	TNF L-selectin CX3CL1 JAM-A	[119,120]

	ADAM17 in leukocytes controls levels of alveolar TNF and L-selectin			

	ADAM17 in endothelial cells regulates adhesion molecules and chemokines			
**Nerve** **recovery**	**Negative role in nerve** **regeneration**	Higher clearance of apoptotic cells and augmented phagocytic capability of microglia	TREM2	[148,150]

	ADAM17 in microglia controls TREM2 levels and phagocytosis			
**Cancer**	**ADAM17 is associated with cancer progression**	Reduced cancer progression	TGFα HB-EGF	[153,156,159, 160]
	
	Regulates EGFR signaling and proliferation of cancer cells	Reduced T cell activation and their ability to kill tumors		
			PD-L1 CD16A	
	Modulates the cleavage of PD-L1 and immuno evasion of cancer cells			
**COVID-19**	**Unclear whether ADAM17 activity is beneficial or detrimental**		ACE2 TNF	[166,167,168]

	Regulates levels of ACE2 and SARS-CoV-2 entry	In vivo models currently not available		
	
	Promotes TNF release and the “cytokine storm”			
**Alzheimer′s Disease**	**ADAM17 has opposite effects in the development of AD depending on its tissue expression**	Decrease of neuroinflammation and phagocytic capability of microglia	TNF TREM2	[135,136,144]

	ADAM17 in microglia regulates TNFR signaling and TREM2 levels	Activation of the anti-amyloidgenic processing of APP and reduced plaque deposition		
			APP	
	ADAM17 in neurons regulates shedding of APP			
**Inflammatory bowel** **disease**	**Protective role in the development of ulcerative colitis**	Diminished EGFR activation, epithelial cell growth and goblet cell differentiation	TGFα	[16,128]

	Controls EGFR signaling			
**Atherosclerosis**	**Protective role in the progression of atherosclerosis**	Enhanced TNFR2 signaling, proliferation of macrophages and their augmented recruitment to the lesion sites	TNF TNFR2	[123]

	Regulates TNFR signaling			

**Table 3:** Inhibitory activity (IC_50_ nM) of selected inhibitors against ADAM17 and ADAM10.

Code	Structure	ADAM17	ADAM10	Ref
**1**	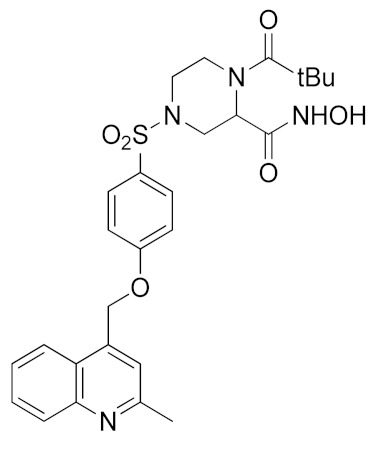	12	>10000	[183]
**2**	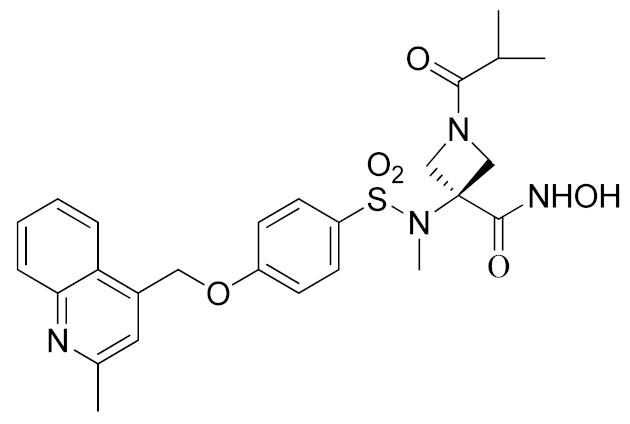	4	950	[184]
**3** (KP475)	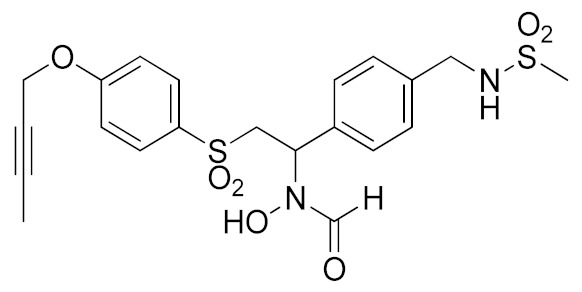	11	748	[185]
**4**	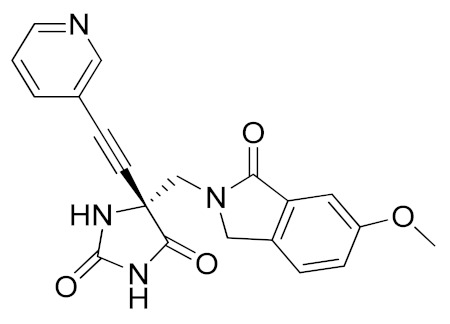	0.62 (*K*_i_)	22	[186]
**5**	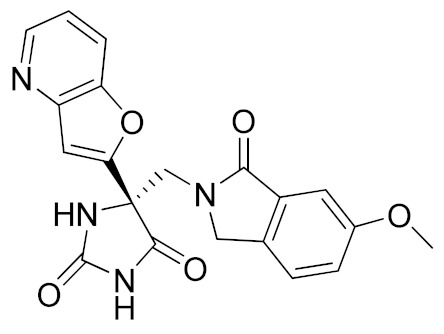	0.5 (*K*_i_)	-*^a^*	[187]
**6**	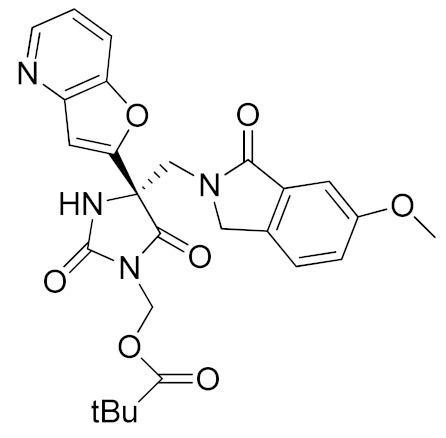	-	-	[188]
**7** (JTP-96193)	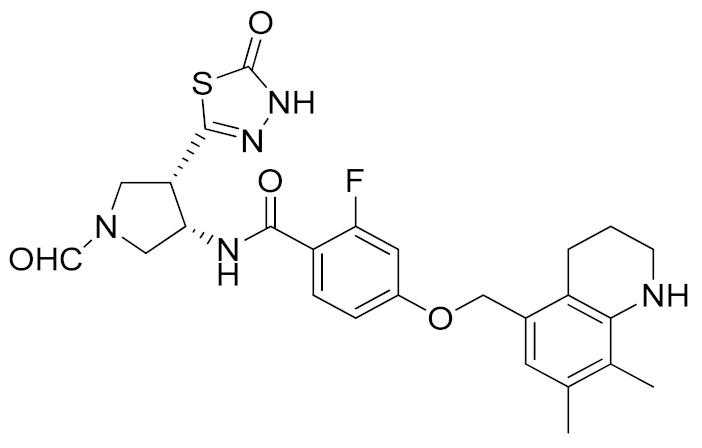	5.4	>10000	[189]
**8**	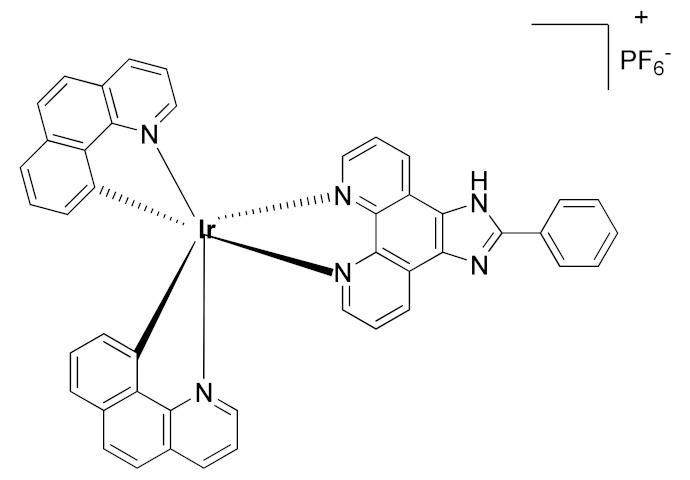	28000	-	[190]
**9**	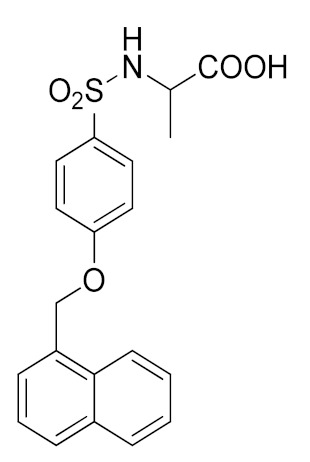	-	-	[191]
**10**	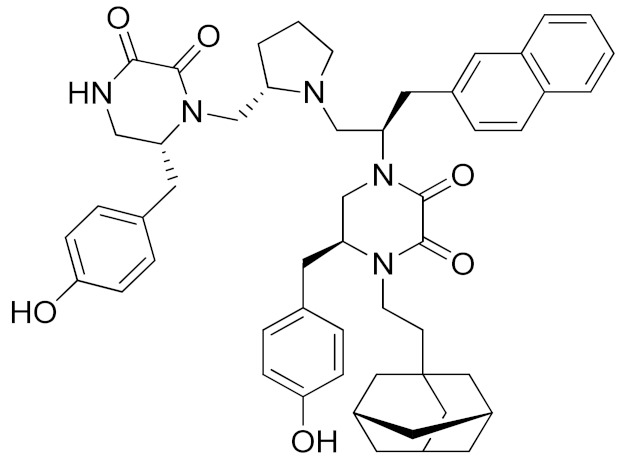	4200	>100000	[192,193]
**11** (ZLDI-8)	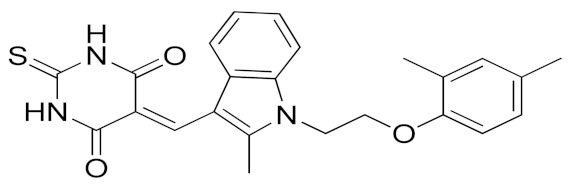	72000	-	[194,195]

*^a^* “-”: Not tested or unknown from the corresponding original reference.

**Table 4:** Reported cell data (IC_50_) and in vivo/ex vivo data for selected ADAM17 inhibitors.

Compound	Effect	Cell Line	IC_50_	In Vivo/Ex Vivo Assay
**1**	Inhibition of TNFα release	Human Keratinocytes (NHEK)	1.3 µM	-
**2**	Inhibition of TNFα release	NHEK	3 nM	Strong activity in a mouse model of oxazolone- induced chronic skin inflammation
**3** (KP475)	Inhibition of GPIbα shedding	human iPSC platelets	≈ 100 nM	Positive results in a thrombus formation model using immunodeficient mice
**5**	Inhibition of TNFα production	human whole blood (hWBA):	287 nM	-
**6**	Inhibition of TNFα production	human whole blood (hWBA):	281 nM	Good PK properties in rats and monkeys
**7** (JTP-96193)	Inhibition of TNF*α* release	rat whole blood (rWBA)	170 nM	In mouse models of obesity, it reduced the TNF-α release from the fat tissue and prevented development of diabetes; in mouse models of diabetes, it improved insulin resistance
**8**	Inhibition of TNF*α* release	PMA-differentiated THP-1 cells	11.24 µM	-
**9**	Inhibition of TNFα release	Rat serum	68.48% inhibition at 20 mg/kg dose	Good anti- inflammatory activity in carrageenan-induced rat paw edema model
**10**	Inhibition of TNF-α cleavage	THP-1 cells	100 µM	-
		
	Inhibition of Heregulin cleavage			
		A549 cells	100% inhibition at 40 µM	
**11** (ZLDI-8)	Inhibition of Notch signaling pathway	LoVo cells	5.57 μM	11 enhanced the effect of Sorafenib on inhibiting tumor growth in a nude HCC-bearing mice model [194]
	
		SW480 cells	7.42 μM	
	
		MHCC97-H cells	5.32 μM	

“-”: Not tested or unknown from the corresponding original reference.

## Data Availability

No new data were created or analyzed in this study. Data sharing is not applicable to this article.
